# Monthly Summary

**Published:** 1887-09

**Authors:** 


### Monthly Summary.

---

TOXIC FFFECTS OF LEAD.—Mr. Wynter Blyth has had an opportunity of examining portions of the bodies of two out of five persons who have at different times died more or less suddenly from, as it is believed, the effects of lead poisoning. In one case he separated about a third of a grain of sulphate of lead from the liver and about a thirteenth of a grain from one kidney, besides finding lead qualitatively in the brain. In the other he was able to examine the brain with more minuteness, and estimated that here the cerebrum contained about a grain and a half and the cerebellum about a quarter of a grain of sulphate of lead. Mr. Blyth went on to remark, in the paper he read to the Chemical Society of London on these investigations: "There has hitherto been no reasonable hypothesis to explain the profound nervous effects of the assimilation of minute quantities of lead, but i it is allowed that lead forms definite compounds with essential portions of the nervous system, it may then be assumed that in effect it withdraws such portions from the body; in other words, the symptoms are produced not by poisoning in the ordinary sense of the term, but rather by destruction—a destruction it may be, of important nerve centres.—*The Lancet.*

---

ONE OBJECTION TO PLASTIC FILLINGS.—Of the cements, it may be truly said that, in approximal cavities especially, they soon become a source of annoyance. However skillfully contoured, they become worn by attrition in a few weeks or months to a degree that allows of food crowding into spaces, or invites contact of the tongue with the slightest projection of enamel edge. To a nervously-constituted person this is a serious annoyance. At the snme time the filling may appear nearly perfect to the eye of the dentist. Only those who, like the writer, have suffered this annoyance, can fully appreciate it.—*Cincinnati Medical and Dental Journal.*

---

THE AMERICAN MEDICAL ASSOCIATION RECOGNIZES DENTISTRY AS A SPECIALTY IN MEDICINE.—*Resolved*: That the regular graduates of such dental and oral schools and colleges as require of their students a standard of preliminary or general education, and a term of professional study equal to the best class of the medical colleges of this eountry, and embraces in their curriculum all the fundamental branches of medicine, differing chiefly by substituting practical and clinical instructions in dental and oral medicine and surgery, in place of practical and clinical instruction in general medicine and surgery, be recognized as members of the regular profession of medicine, and eligible to membership in this association on the same conditions and subject to the same regulations as other members.

Dr. Davis, in introducing the resolution, said he wished to explain its object. There are two objects to be had in view; first, to relieve a degree of embarrassment that exists between the regular profession, as we consider it, and the profession of dentistry. The department of dental and oral surgery is a part of the profession of medicine as much as the department of ophthalmology, or otology, or any other ology. Our teeth and mouths are a part of our system as much as any other part, and are used more than any other part. The embarrassment is this: that in the history of dentistry it was but little, if anything, more than a mere mechanical pursuit. Steadily it has advanced as the years have gone by. A number of years ago, our lamented friend, Prof. S. D. Gross made a proposition that an oral and dental section be provided as a section in this association. It was seconded by Dr. Sayre and myself, and it was organized. The International Medical Congress of 1881 provided a section for dental and oral surgery. The Congress to be held in Washington has done the same thing, and it will be one of the most thorough and best organized sections in the Congress. There is an embarrassment in this respect. It is to know just who and by what line of demarkation those engaged in that department shall be recognized as members of the regular profession. Now it is proposed to make a line and draw it where this resolution says, that all those who are qualified by general education and a term of study equal to the best medical colleges, a curriculum embracing the entire fundamental principles of medicine with the provision that instead of special instruction in clinical surgery, instruction may be had in dental and oral surgery, such shall be recognized as members of the profession of medicine. It will take away a sort of embarrassment. There is more far-reaching and more valuable underlying object in this resolution and that is to be recognized as a member of the profession, if this resolution is adopted by this body, they must have the education received in schools that require these requirements. It makes a strong lever to lift up the course of study in the dental schools. Such are my reasons for bringing up the resolution. I will say nothing more on the subject.

The motion was made that the resolution be adopted by the Association, and it was carried unanimously.

---

TREATMENT OF DIARRHŒA BY IODOFORM AND CHARCOAL.—Picchini treated eight cases of diarrhœa, with symptoms of fermentation, with the following:

| | |
|---|---|
| Iodoform, - - - | grs. 9. |
| Ether, - - - - | ℥ $3\frac{1}{8}$. |
| Vegetable charcoal, finely powdered, | ℥ $3\frac{3}{8}$. |
| Glycerin, ad. - - - | ℥ 12. |

The iodoform must be dissolved in the ether, and the powdered charcoal thoroughly mixed. After the ether has evaporated, the glycerin should be mixed. To take in twenty-four hours, by teaspoonful or tablespoonful, suspended in a glass of water.—*Journal de Medecine.*

HYPNOTIC EXPERIMENTS IN PARIS,—A distinguished company of Parisians recently witnessed some interesting experiments of a newly discovered hypnotiser. M. Moutin does not put people to sleep, but makes them obey his will while thoroughly awake. He began by choosing his subjects among the people who presented themselves, by placing his hand on the nape of the neck. While talking to them he inquired whether they felt an unusual heat under his hand. If an affirmative answer was given he knew he had a good subject, and while telling him to stand up straight, soon brought him on his knees, by simply placing one hand lightly on his back and holding the other in front of his knees. One gentleman, well known in Parisian society, was dragged around the room among the spectators by M. Mouton, who put that gentleman's hand first on his shoulder and then on his head, and told him to follow him. When they got back to the platform he told the same gentleman, when sitting on the ground, that he forbade him to rise. Notwithstanding the most strenuous efforts he could not rise until he had received the magnetiser's permission. One of the writers on the *Gaulois* was operated on in a yet more astonishing manner. Placed at the extremity of the long hall, with his back turned to M. Moutin, he was told to do all he could to prevent himself being drawn backwards toward the platform. He used what seemed to be almost superhuman efforts to stand where he was; but soon his legs began trembling violently, and in spite of all he was soon walking backwards toward the operator. After that everybody was made to laugh heartily by the same gentleman being made to dance in a most amusing manner. M Moutin also fought a mock duel with him. Asking for two walking sticks he gave one to the gentleman, and, after crossing swords with him, paralyzed his arm by his will. After releasing his adversary from that disadvantageous position he was told by M. Moutin that he defied him to touch him with his stick. The operator failed in this instance, for after a prolonged effort, during which the journalist seemed to strain every nerve and muscle in his body, he at last touched M. Moutin's chest. The operator, however, won great applause by re-commencing the experiment. He stood perfectly still, and offered, as before, no resistance, but his will or magnetic power. The gentleman, with his stick, struggled, so to say, against the air, but he failed to touch the operator. One of the ladies present was then told by M. Moutin, while she was sitting among the spectators, that he defied her to say "Nebuchadnezzar." It was ridiculous in the extreme to hear her try in vain, until the operator gave her permission to say the word. The same lady was evidently a good subject, for M. Moutin placing two chairs in the middle of the platform sat down on one, and then told the lady she would come and sit down on the other and lean her head on his shoulder. She protested, but in a few minutes she was seized with a most violent trembling in her outstretched arms. She got up, and then threaded her way amongst the spectators in what seemed to be a nervous trance, for she trembled most violently. Some people thought she would trip on the platform steps, but M. Moutin, who was sitting quietly awaiting her arrival, reassured them by saying, "She cannot fall; I forbid her to fall." She sat down on the chair, and, when there, seemed determined not to put her head on the shoulder of the operator, but in a few moments she closed her eyes aud let her head fall. At the same instant M. Moutin started to his feet, and, blowing in her face, restored her instantly to consciousness. Other equally astonishing experiments were made by him on people whom it is difficult to suppose could be accomplices to a trick.—*Pacific Record of Medicine and Surgery.*

---

SPIDERS.—In spiders the head and thorax are bound together in one piece, and the abdomen connected to it by a very slender neck. Though spiders are provided with hollow poison-fangs, there is no proof that the American species, at least, are poisonous. The jaws of the smaller varieties are too delicate to take hold of the human flesh; and the probability is that even the largest ones are entirely harmless The female spider is the most important member of the family. The males are small and inconspicuous, and in one case a female was found amid the bodies of a large number of her unfortunate husbands, whom she had destroyed and thrown to one side—a perfect female Bluebeard.

The parental love of the spider is very strong. The female carries suspended on her legs, a small bag containing the eggs, which resemble white glass beads. If the bag is pushed away with a straw or stick, the creature will make the most desperate effort to recover it. A spider was once found whose back appeared to have a granulated surface, but upon closer examination showed that she was entirely covered with her young. On trying to shake them off, they attached themselves to their mother by a thread; and on throwing her to the ground, she remained perfectly quiet until they had all pulled themselves back by means of their extemporized cable, and spread themselves over her body as before.

---

A CASE OF REPLANTATION.—In the month of February, a boy ten years old, while playing ball, had the left superior central knocked out; he picked the tooth up and carried it home to show to his father, who, thinking it might grow, put it back in position, but the socket was so torn up that it would not stay. He made the little fellow keep his mouth closed most of the time and kept the tooth in that way for ten days, when he brought the boy to me. I found no union whatever, but much inflammation and suppuration, and quite offensive. I saw at once that it would not be a success in that condition, so I removed the tooth and told him to call again in two weeks. He did so, and I opened the socket, removed the pulp from the tooth, filled the root with lead, and replaced the tooth with splendid results.—*Dr. E. F. Adair.*

MYRTOL.—This is a volatile oil obtained from myrtle leaves and possesses their characteristic odor. Its taste is pungent and slightly acrid, succeeded by a feeling of coolness It is said to be a splendid disinfectant and an energetic antiseptic, to stimulate the digestive functions without upsetting the stomach, and to increase the appetite. In moderate doses myrtol acts as a sedative to the nervous system. It is eliminated by the respiratory and urinary passages. In order to obtain the best results in diseases of the respiratory passages, it should be employed with a view to combating subacute or chronic catarrhal affections, or it may be given at the termination of an acute attack of bronchitis when the fever has subsided. Another indication for its employment is an abundant opaque muco purulent secretion. In these cases the secretion is diminished and rendered less purulent. The average daily dose given is 90 centigrammes in capsules, taken after meals in three doses of 30 centigrammes —*Pacific Record of Medicine and Surgery.*

---

THE ADULTERATION OF CANDIES.—A well-known writer, who has studied, gives the following account of his investigations on this subject: "Terra alba, or white earth, is used largely in the adulteration of candies, The body of candies, the coating of almonds and lozenges are often made from this earthy material. I have seen an ounce of lozenges dissolved in water in which two-thirds of an ounce was white earth. Pine-apple flavor is often obtained from rotten cheese and nitric acid Poisonous coloring is very much used for candies. One of the commonest is "carlot," into which arsenic largely enters. Liquorice drops for the trade are sometimes made of poor brown sugar, glue and lamp-black, flavored with liquorice." It is the duty of mothers to protect their children from the baneful effects of such stuff. The safest way to accomplish this is to keep candies of every kind out of their reach.—*Herald of Health.*

---

PULSE IN MORPHIOMANIA.—Curious sphygmographic tracings obtained in morphio-mania were presented to the French Academy of Sciences in the name of Messrs. Ball and Jennings. The authors point out three types in these tracings: One corresponding to the period of satisfaction, one to the period of desire and one to the period of that peculiar feverish condition resulting from too long a privation of morphine. In all cases a long, flat elevation is observable, following the ascension caused by the systole, and so peculiar that, according to the authors, morphi-nomania might be detected in a person who, for any reason, should try to conceal his vice. It appears therefore that the alcaloid not only leads to a perturbation in the cerebral faculties, but disturbs the functions of all the viscera.

